# Regulatory Roles of MYB Transcription Factors in Root Barrier Under Abiotic Stress

**DOI:** 10.3390/plants15020275

**Published:** 2026-01-16

**Authors:** Arfa Touqeer, Huang Yuanbo, Meng Li, Shuang Wu

**Affiliations:** 1 School of Future Technology & Haixia Institute of Science and Technology, Fujian Agriculture and Forestry University, Fuzhou 350002, China; arfatauqeer1977@gmail.com (A.T.); huangyuanbo65@yeah.net (H.Y.); 2 College of Horticulture, Anhui Agricultural University, Hefei 230036, China; 3 College of Horticulture, Fujian Agriculture and Forestry University, Fuzhou 350002, China

**Keywords:** MYB transcription factors, root barrier, suberin, lignin, abiotic stress

## Abstract

Plant roots form highly specialized apoplastic barriers that regulate the exchange of water, ions, and solutes between the soil and vascular tissues, thereby protecting plant survival under environmental stress. Among these barriers, the endodermis and exodermis play essential roles, enhanced by suberin lamellae and lignin-rich Casparian strips (CS). Recent advances have shown that these barriers are not static structures but are dynamic systems, rapidly adapting in response to drought, salinity and nutrient limitation. The R2R3-MYB transcription factor (TF) family is essential to this adaptive plasticity. These TFs serve as key regulators of hormonal and developmental signals to regulate suberin and lignin biosynthesis. Studies across different species demonstrate both conserved regulatory structure and species-specific adaptations in barrier formation. Suberization provides a hydrophobic structure that limits water loss and ion toxicity, while lignification supports structural resilience and pathogen defense, with the two pathways exhibiting adaptive and interactive regulation. However, significant knowledge gaps remain regarding MYB regulation under combined abiotic stresses, its precise cell-type-specific activity, and the associated ecological and physiological trade-offs. This review summarizes the central role of root barrier dynamics in plant adaptation, demonstrating how MYB TFs regulate suberin and lignin deposition to enhance crop resilience to environmental stresses.

## 1. Introduction

Plant roots deploy specialized apoplastic barriers to regulate the radial movement of water, nutrients, and solutes. These barriers are formed through the localized deposition of hydrophobic polymers, mainly lignin and suberin, within specific root tissues, enabling plants to maintain nutrient homeostasis and stress resilience [1,2,3,4]. The endodermis and exodermis are the root tissues that function as apoplastic diffusion barriers. The endodermis, which separates the cortex from the vascular stele, develops an early lignin-based Casparian strip (CS) during differentiation, followed by the formation of suberin lamellae at later developmental stages. In many plant species, an additional barrier layers the exodermis, differentiates in the outer cortex beneath the epidermis, particularly under stress conditions. Together, these barriers restrict uncontrolled solute transport and protect the root system from environmental challenges [5]. Transcription factors (TFs) are crucial regulators of gene expression in all living organisms, playing significant roles in plant development, cell cycling, cell signaling, and stress response [6]. The development and precise modulation of these root barriers are not static but are dynamically regulated in response to environmental cues. MYB TFs act as integrative nodes that coordinate lignin and suberin biosynthesis with developmental programs and abiotic stress responses [7,8,9].

Specific MYB TFs play essential roles in controlling genes involved in lignin and suberin biosynthesis, transport, and deposition in root tissues, thereby regulating root barrier properties under both normal and stress conditions. Under stress conditions, in response to drought, salinity, and nutrient imbalances, suberization and lignification are increased [10].

However, environmental cues, including nutrient deficiency and hormonal signals, promote suberization without involving CS formation [7]. Suberin formation in the *Arabidopsis thaliana* root endodermis is highly responsive to environmental stimuli, including nutrient deficiency and hormonal signals such as ABA and ethylene, which act antagonistically to maintain suberization homeostasis [3]. TFs play essential roles in regulating both suberin and lignin deposition, coordinating developmental programs with stress-responsive pathways to enhance root barrier function and protect plants against stresses [3]. This review provides current knowledge on the molecular mechanisms, regulatory interactions, and physiological roles of MYB-mediated control of root barrier formation.

## 2. Classification and Diversification of MYB TFs

**Figure 1:**
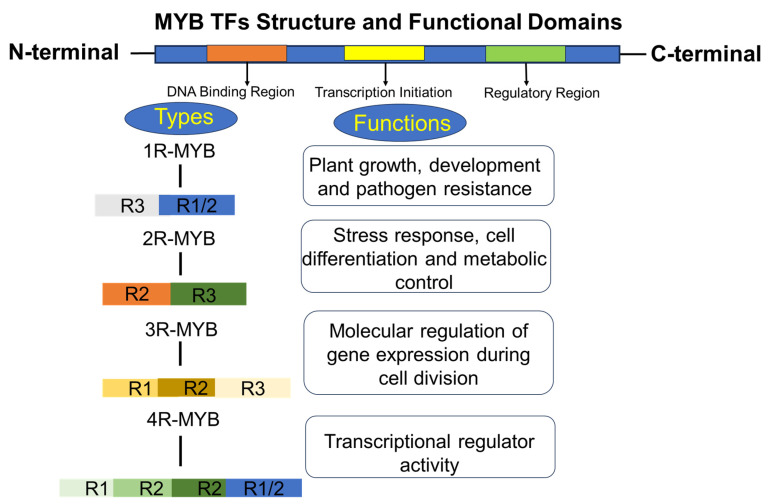
Schematic representation of MYB TFs domain structure, classification, and functions. The diagram illustrates the domain types and associated functions of different MYB subfamilies. The ‘R’ notation refers to the conserved N-terminal DNA-binding domain repeats that define MYB subfamilies, where R1, R2, and R3 indicate different repeat types. Combinations of these repeats (e.g., R1/2, R2R3) characterize distinct subfamilies.

The MYB family represents a large and functionally diverse class of eukaryotic proteins. Members of this family function as TFs and are primarily involved in DNA binding, protein–protein interactions, and the regulation of gene expression [8]. Several MYB proteins have been identified as regulators of diverse cellular processes, including stress responses, development, and cell cycle across different crop species [9]. Based on the number of conserved repeats in their sequences (ranging from 1 to 4), MYB family proteins are classified into four groups: 1R-MYB (one repeat), R2R3-MYB (two repeats), 3R-MYB (three repeats), and 4R-MYB (four repeats). Each repeat comprises three α-helices of approximately 50–53 amino acids, with the second and third helices forming a helix–turn–helix (HTH) motif. This HTH motif contains three equally spaced tryptophan residues that together form a hydrophobic core [11]. The R2R3-MYB subfamily has been further classified into 30–38 groups based on the diversity of conserved domains at the N- and C-terminal regions [12]. The presence and functional roles of MYB TFs have been extensively studied in various plant species, demonstrating their importance as key regulators of biotic and abiotic stress responses (Figure 1). MYB TFs have also been involved in the regulation of suberin biosynthesis, including genes involved in fatty acid modification, glycolipid metabolism, and suberin polymer assembly. Unlike lignin, suberin deposition is highly plastic and responsive to environmental stress, suggesting that MYB TFs contribute to the adaptation of root barriers under adverse conditions [7,8].

### 2.1. R2R3 MYBs in Stress-Induced Suberization

A specialized subgroup of MYBs, functions as master regulators of suberin deposition in response to abiotic stresses. Among these, AtMYB41 is particularly well studied. Its promoter activity is specifically upregulated in suberizing root tissues during stress conditions, including salinity and drought [13,14]. Overexpression of AtMYB44 leads to ectopic suberin accumulation, highlighting its sufficiency in initiating barrier formation. Recent studies further demonstrate that AtMYB41 requires phosphorylation by the MAP kinase MPK6 to activate salt-induced suberin biosynthesis and stress tolerance [14,15]. Likewise, MYB41, MYB53, MYB92, and MYB93 act redundantly but indispensably in mediating endodermal suberin lamella formation. Loss-of-function mutants lacking these MYBs exhibit severely impaired barrier deposition, underscoring their role as central switches in stress-responsive root adaptations [10].

A specialized subgroup of MYBs functions as master regulators of suberin deposition in response to abiotic stresses. Among these, AtMYB41 is particularly well studied; its promoter activity is specifically upregulated in suberizing root tissues under stress conditions, including salinity and drought [13]. Overexpression of AtMYB44 leads to ectopic suberin accumulation, showing its abundance in initiating barrier formation. Recent studies further demonstrated that AtMYB41 requires phosphorylation by the MAP kinase to activate salt-induced suberin biosynthesis and stress tolerance [14,15].

Taken together, abiotic stress signals such as drought and salinity, acting through protein kinase cascades, ultimately activate a network of MYB TFs, each with specialized roles in CS development involving suberin biosynthesis and lignification. The MYB TFs dynamically strengthen root apoplastic barriers, thereby improving water retention, maintaining ionic balance, and regulating oxygen availability under stress. Key MYB TFs involved in root suberin and lignin regulation are summarized in (Table 1).

### 2.2. Hormonal Regulation of MYB-Mediated Stress Responses

Plants regulate stress-related genes and signaling networks to adapt to abiotic stresses [18]. MYB TFs have been observed to be involved in Abscisic Acid (ABA) signaling pathways in response to drought stress. Environmental stresses trigger hormonal signaling that induces the synthesis and deposition of suberin in root barriers. Hormones play distinct roles in regulating root barrier formation under stress. In particular, ABA acts as a strong inducer of suberin biosynthesis, promoting enhanced deposition of suberin lamellae in endodermal cells [20,22]. In rice, an ABA-deficient mutant fails to form the suberized exodermal barrier [23]. Similarly, ethylene regulates suberization under certain nutrient stresses [24]. Suberization is known to increase under conditions such as salt and drought stress, suggesting that suberin functions as a protective barrier that limits the entry of harmful ions and toxic elements into the cytoplasm [25,26,27].

Under drought stress, ABA levels increase in roots, stimulating the accumulation of suberin lamellae in the endodermis, which restricts water loss and helps conserve soil moisture. Similarly, salinity stress induces suberization in the endodermis, which limits sodium influx, thus reducing ionic imbalance in plants [28]. In various crop species and in *Arabidopsis*, approximately 51% of MYB proteins were upregulated (such as AtMYB2/74/102), and 41% were downregulated in response to drought stress [29]. Overexpression of AtMYB44, a member of the R2R3-MYB TF subfamily, has been shown tolerance to drought and salt stress in soybean [30].

Beyond hormone signaling, developmental TFs also govern MYB regulation in barrier formation. SHORT-ROOT (SHR) and SCARECROW (SCR), which specify endodermal cell fate, activate the expression of MYB36 as well as an endodermis-specific microRNA regulatory module. MYB36 is critical for CS differentiation, directly regulating CASPARIAN STRIP MEMBRANE DOMAIN PROTEINS (CASPs), peroxidases, and ESB1, which are required for lignification [31].

## 3. Suberin and Lignin Deposition as an Adaptive Barrier

### 3.1. Biochemical Pathways in Suberin Biosynthesis

Suberin consists of two major domains: an aliphatic polyester matrix and a phenolic domain. It is composed of long-chain (C16–C18) and very-long-chain (C20–C24) fatty acid derivatives, and its biosynthesis requires enzymes from multiple families. Its biosynthesis begins with fatty acid precursors derived from plastidial fatty acid synthesis. Glycerol-3-phosphate acyltransferases (GPATs), particularly GPAT5, catalyze the esterification of glycerol with long-chain and very-long-chain fatty acids to form acylglycerols that constitute the aliphatic suberin domain [32].

Fatty acid modification involves cytochrome P450 enzymes of the CYP86 family, such as CYP86A1 and CYP86B1, which introduce ω-hydroxyl groups into fatty acids, facilitating their subsequent polymerization into suberin polyesters [33]. The biosynthesis of suberin monomers involves both the fatty acid and phenylpropanoid pathways. Fatty acid synthesis takes place in the plastid stroma; however, diffused across plastid membranes. The key steps of suberin biosynthesis occur within the endoplasmic reticulum [27].

**Figure 2:**
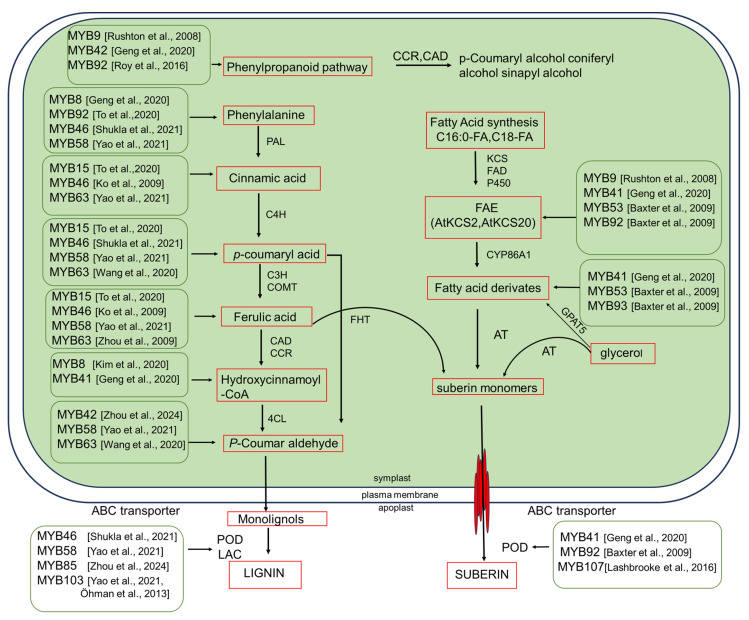
Schematic diagram showing MYB TFs involved in suberin and lignin biosynthesis pathways MYB9 [35], MYB41 [18], MYB99 [11], MYB15 [36], MYB46 [10], MYB58 [37], MYB8 [38], MYB63 [39], MYB42 [19], MYB103 [17,37], MYB85 [40], MYB53 [1], MYB107 [41]. Abbreviations of the proteins are: 4CL, 4-coumarate–CoA ligase; ABC transporter, ATP-binding cassette transporter; AT, acyltransferase; C3H, coumaroyl-CoA 3-hydroxylase; C4H, cinnamate 4-hydroxylase; COMT, caffeic acid O-methyltransferase; FAD, fatty acid desaturase; FAE, fatty acid elongase; KCS, β-ketoacyl-CoA synthase; PAL, phenylalanine ammonia-lyase; P450, cytochrome P450 monooxygenase; POD, peroxidase. (The figure was constructed by integrating information from previously published studies on lignin and suberin biosynthesis pathways) [34,42].

Suberin monomers, once synthesized, are transported from the cytoplasm to the cell wall through ABCG-type ATP-binding cassette (ABCG) transporters. In *Arabidopsis*, ABCG2, ABCG6, and ABCG20 regulate the formation of suberin barriers in roots and seed coats, with triple mutants showing severe abnormalities in suberin lamellae and enhanced tissue permeability [34]. Loss of function in these transporters results in “patchy” suberin deposition and reduced adaptability to drought stress. Together, GPAT4, GPAT6, and GPAT8, which contribute to monoacylglycerol biosynthesis, form a conserved biochemical network that allows roots to dynamically strengthen barriers (Figure 2).

In *Arabidopsis thaliana*, MYB41, MYB53, MYB92, and MYB93 are activated by ABA and act as direct regulators of suberin biosynthetic genes [10]. Root suberization is modulated by abiotic stress conditions and is closely associated with plant stress hormone signaling pathways [3]. Recently, several TFs have been identified as positive regulators of suberin formation across different tissues and plant species [14,41,43,44,45]. AtMYB96 is strongly induced by drought stress through ABA signaling and functions as a molecular hub that integrates ABA and auxin pathways. Specifically, AtMYB96 represses lateral root formation by activating ABI5 and aux-in-responsive genes, thereby reallocating resources toward stress defense rather than growth [30]. This regulation indirectly enhances suberin deposition, strengthening root barriers against water loss and ionic imbalance.

**Figure 3:**
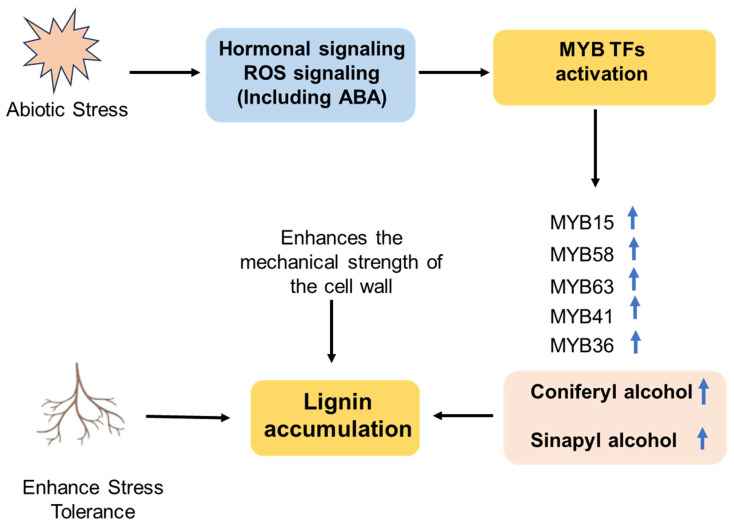
Stress-induced lignin biosynthesis is mediated by MYB TFs. Under stress conditions, plants perceive external signals that trigger hormonal and reactive oxygen species (ROS) signaling pathways. These cascades activate MYB TFs, which mediate stress-induced lignin and suberin biosynthesis. The activated MYB TFs upregulate biosynthetic gene expression, leading to enhanced accumulation of phenylpropanoid precursors in root tissues and increased synthesis of suberin and lignin monomers [14,19,38,39,47].

Auxin also plays a crucial role in MYB-mediated regulation of root stress responses. Experimental evidence shows that auxin promotes AtMYB96 expression in root tissues, establishing a feedback mechanism between developmental growth regulation and stress adaptation. This auxin-ABA interaction fine-tunes MYB96 activity, ensuring that lateral root development is dynamically modulated according drought severity [31]. Many MYB TFs protect plants from different stresses by regulating phenylpropanoid metabolism [21]. This suggests that MYBs function as “stress switches,” mobilizing biochemical pathways in response to water loss or ionic imbalance. Ethylene often antagonizes ABA-induced suberization, whereas jasmonic acid (JA) enhances it, particularly under salt stress [46]. Auxin signaling has also been implicated in the spatial patterning of suberization, although its role under stress remains less well defined. Collectively, these findings indicate that MYBs ensure that suberin deposition is both spatially and temporally coordinated with stress conditions, enabling plants to adapt effectively to adverse environments. Han et al. [47] identified StMYB24, StMYB144, and StMYB168 as key positive regulators of suberin biosynthesis during potato tuber wound healing. StMYB168 activates phenylpropanoid pathway genes, whereas StMYB24 and StMYB144 regulate aliphatic suberin biosynthetic genes. In addition, MYB15 [48], MYB36 [41], and the lignin pathway-activating TFs MYB58 and MYB63 are key regulators that integrate biotic and abiotic signals [19] (Figure 3).

### 3.2. Lignin Deposition and Stress Responses

Lignin is a complex polyphenolic polymer with a biosynthesis pathway from phenylpropanoid precursors that is among the most conserved stress-responsive metabolic pathways in land plants. The pathway begins with the deamination of phenylalanine-by-phenylalanine ammonia-lyase (PAL), followed by hydroxylation of cinnamic acid by cinnamate 4-hydroxylase (C4H), activation by 4-coumarate-CoA ligase (4CL), and subsequent reactions catalyzed by hydroxycinnamoyl transferase (HCT), cinnamoyl-CoA reductase (CCR), and finally cinnamyl alcohol dehydrogenase (CAD) to produce the monolignols (p-coumaryl, coniferyl, and sinapyl alcohols) that polymerize into lignin [37].

Zhou et al. [40] demonstrated that MYB58 and MYB63 function as major transcriptional activators of the lignin biosynthetic pathway during secondary cell wall formation. MYB58 directly binds to AC elements—cis-regulatory motifs in lignin gene promoters and activates target genes, including LAC4, which encodes a laccase involved in lignin polymerization. While the lac11 single-knockout mutant showed normal lignin deposition, the lac11, lac4, and lac17 double-knockout mutants showed a reduction in lignin content [19]. Furthermore, in *Arabidopsis*, MYB46 acts as a transcriptional activator of the LAC11 promoter, according to protoplast transactivation assays [49]. Overall, MYB TFs regulate lignin biosynthesis by activating or repressing the expression of key enzymes in the phenylpropanoid pathway.

### 3.3. Transcriptional Regulation of Lignin

Under stress conditions, lignification occurs in specific root regions as an adaptive modification of the apoplastic barrier. Recent work shows that salinity commonly stimulates enhanced lignification in root tissues, particularly in the stele and cortex, which reduces apoplastic flow and limits the movement of toxic ions to shoots [50]. Combined proteomics, metabolomics, and histochemistry in wheat have demonstrated genotype-dependent activation of phenylpropanoid metabolism and increased monolignol production under salinity; the enhanced lignification correlates with improved root performance under salt stress. Salt stress triggers Ca^2+^ and ROS signaling, as well as specific kinase cascades. These signals integrate into transcriptional networks involving NAC and MYB TFs, which upregulate the expression of phenylpropanoid pathway genes (e.g., PAL, C4H), ultimately leading to increased lignin deposition in root barrier tissues [51]. Under drought or water-deficit conditions, many plants accumulate higher lignin levels. This is primarily driven by the transcriptional upregulation of key phenylpropanoid pathway and monolignol biosynthetic genes [21,31] (Table 2).

Drought causes similar lignification responses, but with tissue- and species-specific patterns. Lignin deposition within xylem and xylem-adjacent cells improves resistance and maintains hydraulic conductivity during water deficit [58]. MYB TFs improve drought tolerance in annual crops, including major cereals, often by stabilizing the xylem and preventing catastrophic loss of hydraulic conductivity [58,59]. NAC and MYB TFs play a key regulatory role in lignin biosynthesis. The OsNAC5 TF activates the rice gene OsCCR10 (Cinnamoyl-CoA Reductase 10), thereby improving drought tolerance at the vegetative stage. Overexpressing plants exhibited increased lignin deposition in roots (H- and G-lignin), enhanced photosynthetic efficiency, and reduced water loss in leaves compared with non-transgenic controls [60]. *OsNAC17* overexpression upregulates multiple lignin biosynthesis-related genes, promotes lignification in both leaves and roots, and thereby strengthens drought tolerance in rice [53]. The regulatory role of MYBs in stress-induced lignification has been explained by several recent studies. MYB15, originally characterized for its role in immune lignification, transcriptionally upregulates PAL, C4H, 4CL, and CAD during a specific immune response to effectors, thereby providing a model to explain how environmental cues activate lignin biosynthetic enzymes [38].

Similarly, MYB46 and MYB83 remain key effectors of NAC-mediated secondary wall formation, but they are also responsive to stress-activated upstream signals [61]. MYB36, although primarily controlling CS formation in the endodermis, regulates the position and pattern of lignin deposition and coordinates adaptive cell wall modifications when barrier integrity is reduced [31,62]. Collectively, these MYB nodes allow plants to spatially and temporally improve lignification in response to specific stresses. MYB15, MYB41, and MYB36, the suberin module, are activated upstream. MYB58 and MYB63 directly upregulate the biosynthetic machinery for monolignols (coniferyl and sinapyl alcohol), leading to lignification. Activated MYB TFs upregulate genes in the phenylpropanoid pathway, leading to increased production of monolignols, such as coniferyl alcohol and sinapyl alcohol, which are polymerized into lignin (Figure 3).

## 4. Stress-Induced MYB TFs in Arabidopsis Compared to Other Species

### 4.1. Tomato (Solanum lycopersicum)

In *Arabidopsis* roots, a focused set of R2R3-MYB TFs controls stress- and hormone-induced endodermal suberization. Four endodermis-expressed MYBs—MYB41, MYB53, MYB92, and MYB93 respond to both ABA signaling and the CS integrity pathway, thereby promoting suberin biosynthesis. Each of these MYB TFs is sufficient to induce suberin deposition in the root endodermis [10]. SGN3 (SCHENGEN3) is a leucine-rich repeat receptor-like kinase localized to the plasma membrane of endodermal cells in plant roots. Its ligands, CIF (CASPARIAN STRIP INTEGRITY FACTOR) peptides, bind to SGN3 to sense defects in the CS and activate signaling pathways that restore barrier integrity by promoting lignin and suberin deposition [10]. Particularly, ABA can induce suberization even in sgn3 mutant backgrounds, indicating that ABA signaling acts independently of, or downstream from the SGN3/CIF pathway. However, CIF peptides can promote suberization when ABA signaling is functional, supporting a model in which developmental cues and stress-related ABA signaling integrate to fine-tune the MYB41/53/92/93 regulatory network, thereby regulating endodermal barrier formation under changing environmental conditions [10,14].

Additional studies further support the involvement of these MYBs in stress-responsive suberization. For example, MYB41 is rapidly induced by drought, salt, and osmotic stress, and its ectopic expression promotes suberin biosynthesis across different tissues. Likewise, MYB92 and MYB93 have been linked to nutrient-deficiency responses, particularly under K^+^ starvation, highlighting their broader role in coordinating developmental and environmental signals in the endodermis [1].

In tomato, a recent study by Cantó-Pastor et al. [63] revealed that SlMYB92 is required for drought-induced exodermal suberization. Genetic disruption of SlMYB92 severely impaired drought tolerance, whereas its over-expression enhanced an exodermal suberin layer that functionally substitutes for the endodermal barrier. Further, it was confirmed that SlMYB41 and SlMYB92 act as positive regulators of exodermal suberin biosynthesis [64].

### 4.2. Rice (Oryza sativa)

A second regulator in *Arabidopsis* is MYB36, which regulates the formation of the lignin-based CS. MYB36 directly activates core CS genes to establish the lignified band that forms a barrier in the endodermis and ensures selective nutrient uptake [21,31]. Loss of MYB36 disrupts CS assembly and barrier function. Notably, *myb36* and other CS-defective mutants often show enhanced suberin in older root zones [65,66]. These MYBs function in a central position at the intersection of developmental and stress signaling. The MYB41/53/92/93 pathway regulates endodermal barrier formation. Because these four MYBs are collectively necessary for full suberization, this design principle helps explain how *Arabidopsis* rapidly enhances root barriers under stress while maintaining developmental control over endodermal differentiation [10,31]. Thus, these findings establish that *Arabidopsis* and rice depend on a core set of stress-responsive. MYBs to regulate suberization and lignification of the endodermis, thereby stabilizing ion homeostasis and improving stress tolerance.

Chen et al. [67] demonstrated that four homologous R2R3-MYB TFs—OsMYB39a, OsMYB41, OsMYB92a, and OsMYB92b—regulate endodermal barrier formation in rice. These OsMYBs function downstream of the CS integrity pathway, which involves OsMYB36a/b/c, OsSGN3, and OsCIF. They integrate both ABA-dependent and stress-induced signaling to promote suberin lamellae formation and lignification in endodermal cells. Functionally, OsMYB39a, OsMYB41, OsMYB92a, and OsMYB92b collectively promote stress-induced suberization and non-polar lignification, thereby enhancing root barriers.

### 4.3. Conserved vs. Species-Specific Mechanisms

For suberization, MYB41 [14], MYB92 [36], and MYB107/MYB9 [41] promote the expression of aliphatic-suberin pathway genes and drive suberin deposition in tissues, indicating a conserved MYB element for suberin biosynthesis. These TFs interact with hormone signaling, particularly ABA, which regulates endodermal suberin in response to exogenous and developmental cues, demonstrating a conserved mechanism of root barriers [10].

In *Arabidopsis* suberin is deposited primarily in the endodermis, where lamellae form a barrier within the root cortex. In contrast, tomato shows both endodermal and exodermal contributions, with suberin-rich lamellae extending to the outer cortex under stress. In cereals, endodermal suberin is enhanced under drought. Together, these findings suggest that while the transcriptional control of suberin synthesis is evolutionarily conserved, the Spatial distribution of suberized barriers emphasize species-specific ecological and structural adaptations. This dual perspective highlights the importance of integrating molecular signaling elements with barrier composition to fully understand the stress-responsive formation of root barriers. Recent research in tomato reveals that a conserved suberin network, driven by SlMYB92, has been modified to function in the exodermis rather than the endodermis, a location where it is essential for drought tolerance [68]. Moreover, the exodermis forms a lignin cap instead of a typical CS, demonstrating species-specific lignin patterning that provides equal barrier function under genetic control [69].

In rice, ABA is required for exodermal suberization that supports the radial oxygen loss (ROL) barrier, which Prevents oxygen in the root from diffusing out too quickly. ABA-deficient mutants fail to form the barrier, while exogenous ABA restores suberin lamellae and barrier function. This finding links stress hormones to exodermal suberin deposition in monocot roots [28].

## 5. Cross-Talk Between Suberin and Lignin Pathways

Roots build two complementary apoplastic defenses: lignin-rich CS (and sclerenchyma) and suberin lamellae to control water, gas, and ion movement. Although these polymers have distinct interactions and cellular locations, they are metabolically and regulatory involved: (1) they draw on shared phenylpropanoid precursors and upstream carbon flows; (2) they are coordinated by overlapping TFs (notably MYBs) that allocate fluxes between the lignin and suberin branches; and (3) they exhibit regulation, such that disruption of one barrier often triggers increased deposition of the other. This review synthesizes recent evidence for each of these aspects and explains how plant MYB TFs coordinate lignification and suberization to maintain barrier formation in changing environments.

### 5.1. Shared Biochemical Precursors and Metabolic Challenges

At the biochemical level, lignin and the aromatic (phenolic) component of suberin share upstream steps in the phenylpropanoid pathway. The conversion of phenylalanine to cinnamic acid by phenylalanine ammonia-lyase (PAL), and subsequent steps through cinnamate 4-hydroxylase (C4H) and 4-coumarate-CoA ligase (4CL), produce hydroxycinnamoyl-CoA esters that serve as substrates for both monolignol biosynthesis and the production of phenolic suberin constituents [18,70]. At the aliphatic level, suberin requires fatty-acid-derived monomers (ω-hydroxyacids, α,ω-diacids), produced by fatty-acid modification enzymes (CYP86 family, FAR5, GPAT5). A biosynthetic branch largely distinct from lignin synthesis [32,71]. However, the aromatic domain of suberin (hydroxycinnamates and feruloyl esters) draws directly from phenylpropanoid intermediates [70]. Although the interactions of suberin’s aliphatic and lignin’s phenolic polymers are distinct, they converge in phenylpropanoid metabolism. This creates a metabolic “junction” where allocation decisions are made. Coordinated control of enzyme activity and precursor distribution ensures that plants maintain barrier integrity while balancing energy under developmental or environmental limitations.

### 5.2. MYBs as Decision Nodes Balancing Lignin and Suberin

Transcriptional control is essential in phenylpropanoid metabolism. MYB TFs govern secondary wall formation and lignification, and their role is well established in xylem development. MYB activators MYB46 and MYB83 act as a second-tier regulatory hub in secondary cell wall biosynthesis, activating downstream TFs such as MYB20, MYB42, MYB43, and MYB85 through direct binding to the SMRE (Secondary Wall MYB-Responsive Element), a specific cis-regulatory DNA sequence bound by secondary cell wall-associated MYB TFs (e.g., MYB46 and MYB83) to activate downstream genes involved in cell wall biosynthesis [72,73]. MYB58 and MYB63 [74], MYB103 [17] function as transcriptional regulators that directly promote lignin biosynthetic gene expression during secondary wall formation in *Arabidopsis*. In contrast, MYB4, MYB7, MYB32, and MYB75 act as negative regulators that repress lignin biosynthesis Significantly, recent work in *Arabidopsis* has shown that many MYBs function as central nodes within these regulatory networks; some MYBs activate suberin genes while repressing lignin genes, others promote CS formation and lignification (e.g., MYB36) [20]. Some R2R3-MYB proteins, such as AtMYB58 and AtMYB63, positively regulate lignin biosynthesis in the cell wall [19].

This provides plants with flexible “switches”. For example, ABA-induced activation of suberin-promoting MYBs enhances suberization by upregulating GPAT5 and CYP86s, while downregulating genes necessary for localized CS assembly [10,20]. However, when developmental programs require a CS (e.g., during endodermal differentiation), MYB36 and its downstream associates enhance lignin biosynthesis and spatially restrict suberin deposition until later developmental stages [20,31].

### 5.3. Compensatory Regulation: Loss of One Barrier Amplifies the Other

Endodermal differentiation is regulated by a transcriptional network that includes SHORT-ROOT (SHR), SCARECROW (SCR), and MYB36 key TF involved in root development, particularly in the formation and patterning of the endodermis in plants [50,75]. MYB36 regulates the expression of key genes required for CS formation, including Casparian strip membrane domain proteins (CASPs), Enhanced Suberin1 (ESB1), and Peroxidase 64 (PER64) [21,31].

Reyt et al. [66] showed that defects in nanodomain formation at the CS are associated with increased suberin deposition in neighboring zones, providing evidence that the cell regulates CS integrity and triggers barrier support. Xu et al. [20] and Shukla et al. [10] further identified transcriptional pathways that explain this response: sensing pathways (CIF peptides, SGN3 receptor kinase, and SGN1 signaling) detect CS defects and activate downstream MYBs and peroxidases, thereby activating both lignin deposition and suberin biosynthesis depending on the condition.

Several factors determine whether plants favor lignin or suberin when transcriptional capacity is limited. The key factors are: (i) local developmental cues (SHR/SCR/MYB36 axis specifying CS domain); (ii) hormonal signals, especially ABA, which promote suberization under water or ionic stress; (iii) peptide sensing via the CIF–SGN module, detects defects in the CS and activates repair responses [10,20,70].

**Figure 4:**
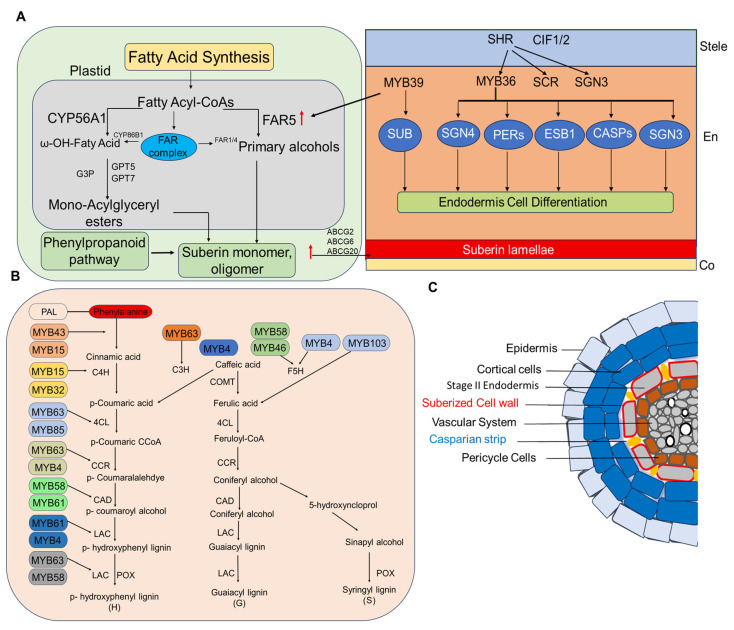
Suberin and lignin biosynthesis: (**A**) Suberin Biosynthesis. The interaction between CS-related genes promotes the formation of suberin lamellae [26,50,77]. (**B**) Lignin biosynthesis pathway showing key enzymes and their regulation by specific MYB TFs [18,19,38,62,78,79,80]. Abbreviations: PAL, phenylalanine ammonia-lyase; C4H, cinnamate 4-hydroxylase; C3H, p-coumarate 3-hydroxylase; COMT, caffeic acid O-methyltransferase; 4CL, 4-coumarate:CoA ligase; CCR, cinnamoyl-CoA reductase; CAD, cinnamyl alcohol dehydrogenase; F5H, ferulate 5-hydroxylase; LAC, laccase; POX, peroxidase; H lignin, p-hydroxyphenyl lignin; G lignin, guaiacyl lignin; S lignin, syringyl lignin (**C**) Cross-section of a plant root showing the endodermal root barrier; yellow ovals represent CS, and red indicates suberin encasing endodermal cells at the primary growth stage.

Li et al. [50] reported that root suberization is closely associated with CS-synthetic gene groups, including SHR, SCR, MYB36, SCHENGEN (SGN) 3, SGN4, ENHANCED SUBERIN 1 (ESB1), peroxidases (PODs), Casparian strip membrane domain proteins (CASPs), and (CIF) Casparian strip integrity factor. MYB39, which plays an important role in CS formation, is linked to the promoter of FAR5, a gene involved in suberin biosynthesis and suberin deposition in the root endodermis [7] (Figure 4A). Among the genes encoding suberin-biosynthetic enzymes, fatty acyl-CoA reductases have been identified in *Arabidopsis* roots [69]. ABC transporters play a direct role in the transport of suberin polyesters, as demonstrated in the *Arabidopsis* abcg2 abcg6 abcg20 triple mutant upregulated, which exhibits distinct structural changes in root suberin [34] (Figure 4A). Lignin monomers can diffuse freely within the cell, but their polymerization sites are determined by immobilized oxidases that restrict their movement. Laccases (LACs) and class III peroxidases (POXs) catalyze the oxidative polymerization of these monomers. Through the activity of POXs and LACs, the monolignols polymerize to form three main types of lignin: p-hydroxyphenyl (H), guaiacyl (G) and syringyl (S) lignin [65] (Figure 4B). MYB85 was shown to activate the expression of the lignin biosynthetic gene *4CL* in a transient expression assay using *Arabidopsis* protoplasts [76] (Figure 4B). In summary, MYBs provide both activator and repressor functions that transfer transcriptional resources between lignin and suberin branches in response to developmental and environmental inputs. MYB TFs regulate lignification by controlling the expression of enzymes in the phenylpropanoid pathway [74] (Figure 4B). Suberin deposition in endodermal cell walls, together with CS formation, creates a selective root barrier that limits apoplastic transport and controls solute transport into the vascular system (Figure 4C).

## 6. Evolutionary and Comparative Perspectives

Phylogenomic studies show that the R2R3-MYB TF family expanded early in land-plant evolution and subsequently diversified into subfamilies with conserved DNA-binding domains but highly divergent C-terminal regulatory regions that specify function [81]. This expansion created a large set of paralogs that were subsequently functionalized across families. This allowed for the independent recruitment of MYB family members into diverse metabolic programs, including suberin and lignin biosynthesis, as well as flavonoid production [82]. Genome-wide surveys in cereals, dicots, and woody species consistently identify approximately 100 R2R3-MYBs per genome, with clear orthologs in many subfamilies [83,84]. The practical implication is that a broadly conserved MYB TF network exists across angiosperms. This enables equivalent regulatory logic, such as the stress-induced activation of genes like GPATs or PAL—even when the specific MYB paralog integrated into differs between species.

**Figure 5:**
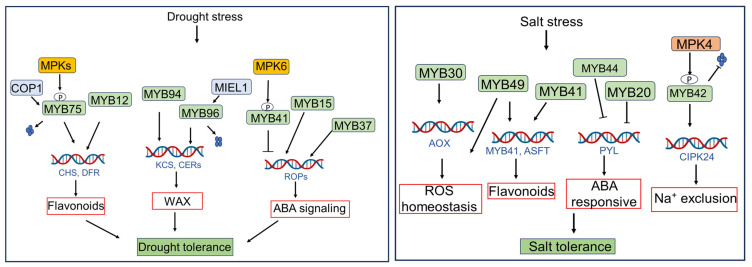
Regulatory roles of MYB TFs in plant responses to drought and salt stress. MYB TFs control the expression of biosynthetic genes involved in the production of metabolites, such as flavonoids and waxes, through ABA signaling, thereby enhancing plant tolerance to drought and salt stress. MYB44 and MYB20 positively regulate plant salt tolerance [91,92]. MYB30 promotes the expression of genes that maintain cellular redox homeostasis, while MYB49 regulates antioxidant defenses [93]. while MPK4 phosphorylates MYB42 to protect it from degradation under salt stress [94]. Abbreviations: MPKs, Mitogen-Activated Protein Kinases; COP1, Constitutively Photomorphogenic 1; MYB, Myeloblastosis transcription factor; KCS, β-Ketoacyl-CoA Synthase; CERs, Eceriferum proteins; ROPs, Rho-like GTPases of Plants; MIEL1, MYB30-Interacting E3 Ligase 1; ASFT, Alcohol-forming Fatty Acyl-CoA Reductase; ROS, Reactive Oxygen Species; CIPK24, CBL-Interacting Protein Kinase 24; Na^+^, sodium ion.

The same upstream signals (for example, ABA accumulation under drought) can induce different MYB paralogs in different species or tissues, producing suberin lamellae in one species and enhanced lignification in another without requiring extensive adaptation of the pathway. In addition, both MYB46 and MYB83 can directly bind to the promoters of their target biosynthetic genes to enhance lignin precursor production [85]. Overexpression of MYB12 and MYB75 in transgenic plants promotes the accumulation of flavonoids with strong antioxidant activity, thereby enhancing tolerance to abiotic stresses, including drought [86,87] (Figure 5).

Biochemical studies have identified two main types of suberin precursors: 2-acylglycerol-3-phosphates synthesized in the endoplasmic reticulum and aliphatic hydroxycinnamate synthesized in the cytosol [88]. MYB15 and MYB37 act as positive regulators in the plant response to ABA and drought stress. Their expression is strongly induced by ABA and the overexpression of either gene enhances drought tolerance by promoting ABA-mediated responses [89,90]. MYB20 confers salt tolerance by directly binding to the promoters of PYL genes, key negative regulators of ABA signaling, and repressing their expression [91] (Figure 5). MYB TFs contribute to plant salt tolerance through diverse mechanisms. For example, MYB49 enhances suberin and wax deposition barriers for ion exclusion by directly activating biosynthetic genes such as MYB41, ASFT, CYP86B1, and FACT. Furthermore, MYB49 enhances cellular defenses by increasing cytosolic Ca^2+^ levels and upregulating antioxidant genes, including those encoding peroxidases [92] Overexpression of MYB12 and MYB75 in transgenic plants significantly increased the accumulation of flavonoids with strong antioxidant activity, thus leading to enhanced tolerance to abiotic stresses such as drought and oxidative stresses [87] (Figure 5).

## 7. Conclusions and Future Perspectives

TFs play pivotal roles at the transcriptional level by either suppressing or activating genes under diverse stresses. Approximately 7% of the coding capacity of the vascular plant genome is devoted to TFs, which regulate gene expression at the transcriptional level [35]. MYB TFs function as a defense against different stress conditions by regulating suberin and lignin deposition. Collectively, the evidence demonstrates that root barriers are not fixed structures but flexible, highly plastic systems continuously modified by developmental cues and abiotic stress.

At the molecular level, the deposition of suberin and lignin is regulated by a network of hormonal and transcriptional signals. ABA emerges as the dominant activator of stress-induced suberization, particularly under drought and salinity. Ethylene and jasmonic acid act in antagonistic ways depending on the stress context. MYBs such as MYB41, MYB53, MYB92, and MYB93 in Arabidopsis, along with their homologs in rice and tomato, act redundantly yet their combined function is essential for stress-induced barrier formation.

In conclusion, the relationship between suberin and lignin barriers, regulated by MYB TFs, demonstrates the complexity of plant adaptive strategies. These barriers are not passive filters but active, stress-responsive systems that mediate survival at the soil–plant interface. Although TFs as mediators of stress have been used to produce stress-tolerant plants, advanced gene-editing technologies such as CRISPR/Cas9 are powerful tools that can be explored in the future to engineer crops with improved tolerance to drought and salinity. The ultimate success of these efforts will depend on addressing key gaps: understanding barrier regulation under stress combinations, resolving cell-type-specific networks at single-cell resolution, and carefully balancing resilience with growth. From molecular, physiological, evolutionary, and applied perspectives, future research on root barriers has the potential not only to deepen our fundamental understanding of plant resilience but also to deliver practical solutions for sustainable agriculture.

## Figures and Tables

**Table 1:** Key MYB TFs in suberin and lignin regulation.

Genes	Expression	Phenotypes	References
MYB103	Lignifying Tissues(Stem, leaves and flowers)	Enhanced lignification	[16,17]
MYB31 MYB42	Lignifying tissues(Stem, leaves and flowers)	Reduced lignification	[18]
MYB58 MYB63	Secondary differentiation	Abnormal lignification	[19]
MYB85 MYB20 MYB42 MYB43	Lignifying tissue(Stem, leaf, root and flower)	Enhanced lignification	[18]
MYB74	Suberin deposition	Enhanced suberization	[20]
MYB36	Suberized tissues(Root endodermis)	Enhanced suberization	[21]
MYB46/85	Lignifying Tissues (Root and stem)	Enhanced Lignification	[22]

**Table 2:** Effects of different stress treatments on lignin accumulation in various plant species.

Species	Treatment	Lignin Accumulation	Tissues	References
*Arabidopsis thaliana*	1 µM ABA	Increased	Roots (Inner metaxylem position and cortical cell)	[3,39]
*Zea mays*	PEG6000	Increased	Root (xylem fiber)	[52]
*Oryza sativa*	16%PEG6000	Increased	Stem and roots	[53]
*Cucumis melo*	8% PEG6000	Reduced	Stem and roots	[54]
*Cicer arietinum*	Holding water	Increased	Roots (meta xylem and protoxylem)	[55]
*Camellia sinensis*	Holding water	Increased	Non-identified	[56]
*Malus domestica*	Holding water	Increased	Roots	[48]
*Vitis vinifera*	PEG6000	Increased	Secondary xylem	[57]

## Data Availability

The original contributions presented in this study are included in the article. Further inquiries can be directed to the corresponding author(s).
